# Distant ureteric metastasis of prostate cancer four years post radical prostatectomy

**DOI:** 10.1016/j.eucr.2025.102949

**Published:** 2025-01-15

**Authors:** Sachin J. Joshi, William J. Yaxley, Devang J. Desai

**Affiliations:** a St Vincent's Hospital Toowoomba, 22-36 Scott St, Toowoomba City, QLD, 4350, Australia; b University of Queensland, Toowoomba Regional Clinical Unit, 152 West St, South Toowoomba, QLD, 4350, Australia; c University of Southern Queensland, UniSQ Toowoomba, 487-535 West St, Darling Heights QLD, 4350, Australia; d Griffith University, 170 Kessels Road, Nathan, QLD, 4111, Australia

**Keywords:** Ureter, Metastasis, Prostate cancer, Surgery, Hormone therapy, Chemotherapy

## Abstract

We describe a case of a 78-year-old male with a history of Gleason score 9 prostate cancer treated with a robotic-assisted radical prostatectomy, who developed symptoms of right ureteric obstruction four years later.

Diagnostic evaluation revealed right sided hydroureteronephrosis on imaging. Further correlation with prostate specific antigen (PSA) and histopathology from a distal ureterectomy with reimplantation revealed metastatic prostate cancer as the cause of obstruction with incidental focal carcinoma in situ (CIS) also identified.

This case highlights the diagnostic challenges and management strategies for ureteric metastasis of prostate cancer and contributes to the limited body of literature on such cases.

## Highlights

• Prostate cancer can metastasize to the ureter years after primary treatment, challenging diagnosis.
• Differentiating ureteric metastasis from primary urothelial carcinoma requires thorough evaluation.
• Initial benign biopsy results may miss metastatic disease; surgical intervention can be crucial.
• Persistent PSA monitoring post-prostatectomy aids in detecting late metastasis and recurrence.
• A multidisciplinary approach improves outcomes in rare metastatic prostate cancer cases.

## 1 Introduction

Prostate cancer is one of the most prevalent malignancies affecting men, particularly in men over the age of 65. The majority of cases are diagnosed when the disease is localised to the prostate.^1^ However, a significant number of cases progress to advanced metastatic disease, necessitating aggressive treatment strategies including surgery, radiation, and systemic therapies. Prostate cancer has the potential to metastasize to various organs, with common sites including bones, lymph nodes, lungs, and liver. Metastasis to the ureter is exceedingly rare, with fewer than 50 documented cases in the literature.^1^, ^2^, ^3^

The clinical presentation of ureteric metastasis from prostate cancer often mimics that of primary urothelial carcinoma or benign conditions such as ureteral strictures. Patients can present with symptoms of ureteral obstruction, such as flank pain, haematuria, and hydronephrosis.^4^ The rarity of ureteric metastasis from prostate cancer necessitates a high index of suspicion and thorough diagnostic evaluation to differentiate it from primary ureteral tumours and other benign conditions.

We present the case of a 78-year-old gentleman with a history of Gleason score 9 prostate cancer, previously treated with a radical prostatectomy, who presented four years after his initial surgical management with symptomatic right-sided distal ureteric obstruction and hydroureteronephrosis. Subsequent endoscopic assessment demonstrated an obstructive distal ureteric lesion on ureteroscopy which was suggestive of a ureteric urothelial carcinoma, as well as an incidental finding of two bladder tumours near the right ureteric orifice. A distal ureterectomy was performed and histopathology demonstrated metastatic prostate cancer as the cause of ureteric obstruction, with incidental focal CIS also found in the ureter. This case report contributes to the limited body of literature on this uncommon metastatic pathway, underscoring the importance of considering metastatic prostate cancer in the differential diagnosis of ureteral obstruction, in patients with a history of prostate cancer.^2^^,^^5^

## 2 Case presentation

A 78-year-old male presented to his primary health provider complaining of constant right iliac fossa pain, occurring on a background of Gleason score 9 prostate cancer having been treated with a robotic-assisted radical prostatectomy four years prior. Serum biochemistry demonstrated an eGFR of 52, reduced from a previous level of 83 four years prior. A Computed Tomography (CT) scan of the abdomen and pelvis with portal venous contrast was performed and demonstrated moderate right-sided hydroureteronephrosis (Figure A). This was secondary to a 13 × 6mm intraluminal obstructive lesion identified 2cm proximal to the vesicoureteral junction, while the bladder, contralateral ureter and kidney appeared normal. His most recent PSA level ∼3.5 months prior to presentation was 0.16, after previously being unrecordable (<0.008) for over three years.

**Figure A:**
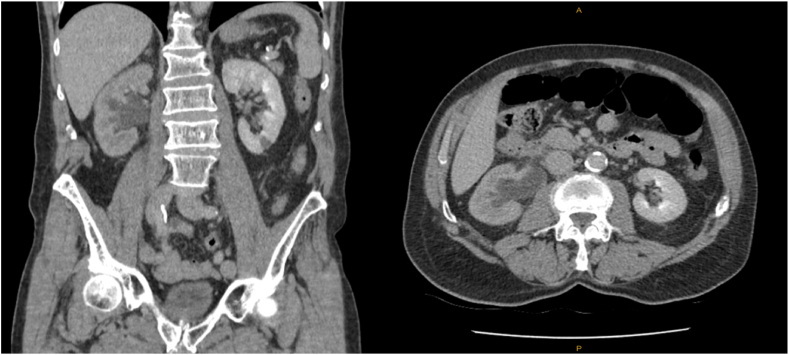
CT Abdomen/Pelvis demonstrating right -sided hydroureteronephrosis.

The patient was referred to a urologist, and further investigation with cystoscopy and uretereoscopy was performed. Cystoscopy revealed an incidental finding of two bladder tumours close to the right ureteric orifice. These were removed and sent for pathological analysis which demonstrated high-grade non-invasive urothelial carcinoma (HGTa). An intraoperative right retrograde pyelogram demonstrated a visible filling defect in the distal right ureter (Figure B). Right ureteroscopy revealed an obstructive mucosal lesion in the distal right ureter which was biopsied, and a ureteric stent was placed at the end of the case (Figure C, Figure D). The biopsy of the circumferential distal ureteric lesion was reported as benign.

**Figure B:**
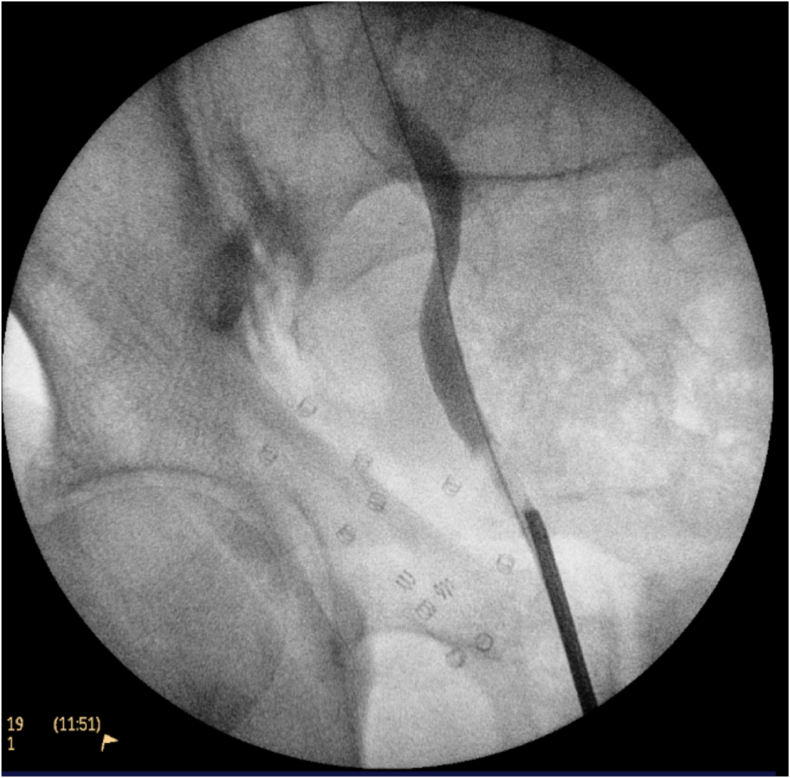
Intraoperative Retrograde Pyelogram demonstrating right distal ureteric filling defect.

**Figure C:**
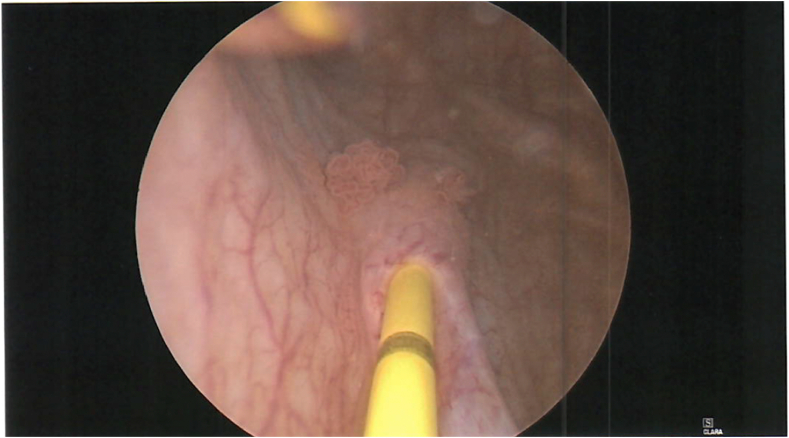
Cystoscopy demonstrating bladder tumours near right ureteric orifice.

**Figure D:**
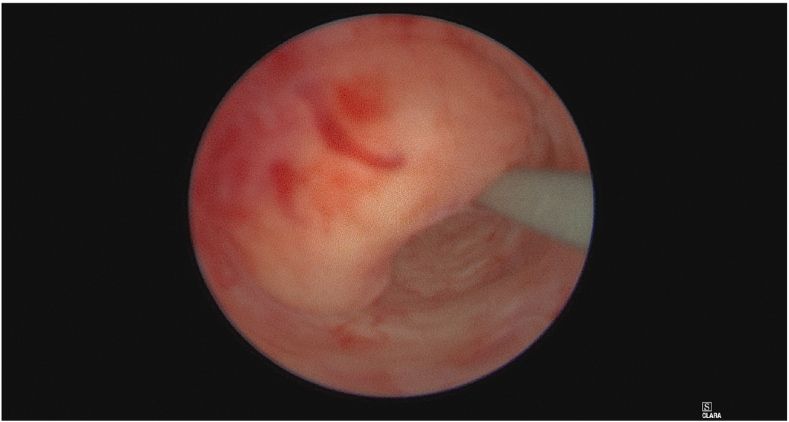
Ureteroscopy demonstrating obstructive intraluminal mucosal lesion in the distal right ureter.

Due to demonstrable obstruction on both CT and intraoperative imaging, as well as histopathology demonstrating urothelial carcinoma of the bladder, a distal ureterectomy and reimplantation of the right ureter was recommended and subsequently performed. Histopathological analysis of the resected ureter revealed metastatic high-grade prostate adenocarcinoma and incidental focal CIS. This was confirmed on immunohistochemical staining, which was diffusely positive for AE1/AE3 and NKX3.1 and showed focal positivity for CK 20 and MUC5ACA in the glandular-like area. There was evidence of neuroendocrine differentiation with synaptophysin and CD 56 positivity. However, the overall impression upon examination by a uropathological specialist remained metastatic prostatic adenocarcinoma with focal carcinoma in-situ (Figure E).

**Figure E:**
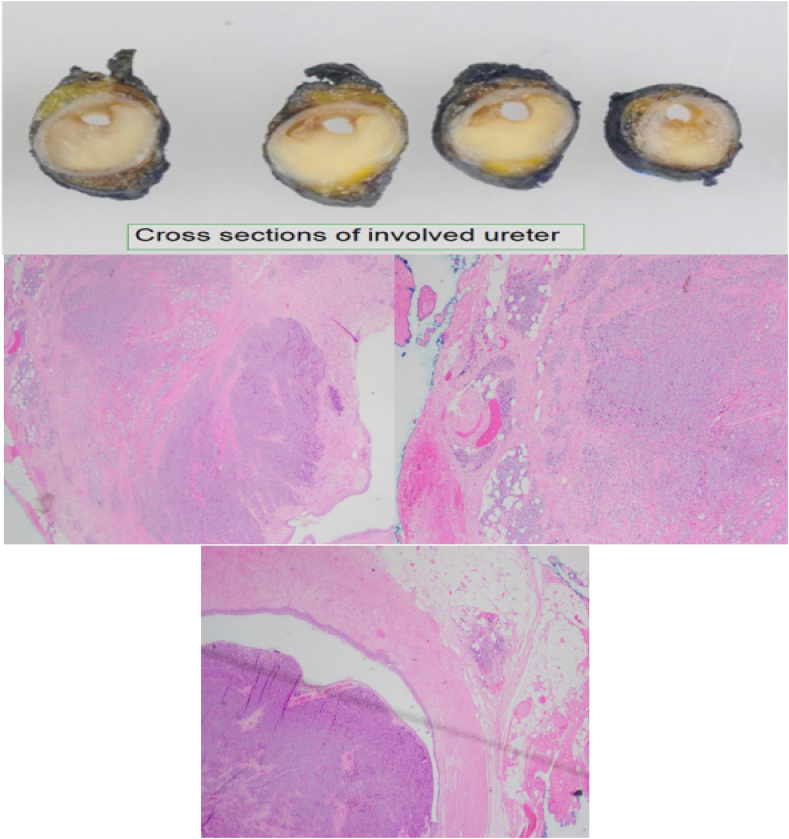
Macro- and Microscopic appearance of the resected right ureter.

A prostate-specific membrane antigen positron emission tomography CT (PSMA PET/CT) scan was completed approximately three weeks post-operatively, revealing multiple regional lymph node metastases in the right internal and external iliac nodes, as well as distant nodal metastases in the right common iliac, retroperitoneal, mediastinal and left medial supraclavicular regions. Multiple skeletal metastases were identified. The PSA was also noted to rise in the initial post-operative setting up to 2.4 μg/L. The patient was referred to medical oncology who subsequently commenced androgen deprivation therapy, docetaxel, and darolutamide. The patient demonstrated an initial PSA response, down-trending to a level of 0.02 μg/L at roughly four months after diagnosis of metastatic disease, before rising to 0.043 μg/L two months later. Six months post-operatively, an interval PSMA PET/CT scan demonstrated radiological disease progression with an increased burden of metastatic deposits. The patient remains under the care of oncologv services.

The patient was also noted to have an improvement in renal function after his procedure, with his eGFR rising to a level of 72 approximately five months after his distal ureterectomy and ureteric reimplantation. From a symptom perspective, there was also a complete resolution of his right iliac fossa pain after surgical management. Surveillance flexible cystoscopy was conducted nine months after distal ureterectomy and reimplantation, revealing no evidence of urothelial carcinoma recurrence. There were no signs of recurrence or obstruction observed on delayed phase CT imaging of the kidneys, ureters, and bladder. Urine cytology also returned negative results for high-grade urothelial carcinoma in three samples.

## 3 Discussion

Ureteric metastasis from prostate cancer is exceedingly rare, making diagnosis and management challenging. The patient's presentation with distal ureteric obstruction in the setting of a simultaneous bladder cancer diagnosis led to a high suspicion of ureteric urothelial carcinoma. However, in this incredibly rare case, metastatic prostate cancer was proven as the cause of the obstruction, with an incidental finding of focal CIS also found in the ureteric specimen. To our knowledge, a case of metastatic prostate cancer to the ureter causing obstruction, with concurrent diagnoses of focal ureteric CIS and HGTa urothelial carcinoma of the bladder has not yet been demonstrated in the literature.

The initial benign biopsy of the ureteric lesion illustrates the difficulty in diagnosing metastatic prostate cancer based on limited tissue sampling. Clinical suspicion of malignancy leading to subsequent surgical management, and histopathological analysis following ureterectomy were crucial in confirming the diagnosis. This aligns with findings from Chung et al., emphasising the necessity of thorough diagnostic evaluation in similar cases, and ensuring that clinical judgment is used in conjunction with investigation results to avoid missing recurrent metastatic disease.^4^

Another important learning point from this case is the importance of post-operative PSA monitoring following radical prostatectomy. This patient presented with symptomatic metastasis four years following resection, with PSA initially rising at three years post-op. The rare occurrence of ureteric metastasis, as seen in this case, highlights an atypical metastatic pathway that clinicians should be aware of. This reiterates the diagnostic challenge reported in the literature in identifying ureteral metastasis using imaging alone, suggesting that completing a comprehensive workup with PSA, cystoscopy/ureteroscopy and/or pyeloscopy and biopsy of any suspicious lesions is warranted.^3^ This should also be guided by clinical suspicion.

Overall management of ureteric metastasis from prostate cancer involves addressing both the obstructive uropathy and the underlying metastatic disease. In this case, given the size of the ureteric lesion, its circumferential nature and its demonstrated subsequent obstruction of the right-sided upper urinary tract, a distal ureterectomy and ureteric re-implantation was considered the most appropriate option, as opposed to other management options for ureteric lesions such as endoscopic ablation. The European Association of Urology guidelines on upper urinary tract urothelial carcinoma management support the consideration of endoscopic ablation in cases of localised low-risk upper tract urothelial carcinoma. However, in cases of high-risk localised urothelial carcinoma, surgical management is recommended either with radical nephroureterectomy or distal ureterectomy ± lymph node dissection ± peri-operative chemotherapy.^6^ The initial benign ureteric biopsy and histopathological diagnosis of bladder HGTa raised the suspicion of a ureteric urothelial carcinoma causing right-sided ureteric obstruction. However, a distal ureterectomy and ureteric re-implantation was crucial to establishing a histopathological diagnosis of ureteric metastasis of prostate cancer with focal CIS. Furthermore, it successfully relieved the patient's ureteric obstruction, with improved renal function and resolution of symptoms post-operatively. Subsequent hormone and chemotherapy aims to control nodal and skeletal metastatic disease, consistent with recommendations for advanced prostate cancer treatment.^5^^,^^7^

## 4 Conclusion

Metastatic prostate cancer to the ureter is rare but should be considered in patients with a history of prostate cancer presenting with ureteral obstruction. Timely and thorough diagnostic evaluation and appropriate management are crucial for optimal patient outcomes. This case highlights an extremely rare case of metastatic prostate cancer to the ureter, with additional incidental simultaneous findings of focal ureteric CIS and high-grade non-muscle invasive urothelial carcinoma of the bladder. It underscores the importance of maintaining a high index of suspicion for metastatic disease in similar clinical scenarios.^5^^,^^7^ It contributes to the limited body of literature on ureteric metastasis from prostate cancer.

## CRediT authorship contribution statement

**Sachin J. Joshi:** Writing – review & editing, Writing – original draft. **William J. Yaxley:** Writing – review & editing, Supervision. **Devang J. Desai:** Supervision, Conceptualization.

## Conflicts of interest

The authors declare no conflicts of interest.

## Abbreviations

CT: Computer Tomography
CIS: Carcinoma in situ
PSA: Prostate Specific Antigen
PET: Positron emission tomography
PSMA: Prostate surface marker antigen
